# Components of metabolic syndrome increase the risk of mortality in nonalcoholic fatty liver disease (NAFLD)

**DOI:** 10.1097/MD.0000000000010214

**Published:** 2018-03-30

**Authors:** Pegah Golabi, Munkhzul Otgonsuren, Leyla de Avila, Mehmet Sayiner, Nila Rafiq, Zobair M. Younossi

**Affiliations:** a Betty and Guy Beatty Center for Integrated Research, Inova Health System; b Center for Liver Disease, Department of Medicine, Inova Fair, Falls Church, VA.

**Keywords:** metabolic syndrome, mortality, NAFLD

## Abstract

Nonalcoholic fatty liver disease (NAFLD) is the most common cause of chronic liver disease in the United States. Metabolic syndrome (MS) components are highly prevalent in NAFLD. Our aim is to assess the relationship of NAFLD and MS with long-term outcome of mortality.

The Third National Health and Nutrition Examination Survey (NHANES) was utilized. NAFLD was diagnosed by ultrasound in the presence of hepatic steatosis and no other causes of chronic liver disease. History of MS and its components were obtained from self-reported NHANES questionnaires. Mortality was obtained from Mortality-Linkage File, through December 31, 2011. Chi-square test was used for categorical variables and Cox proportional models estimated hazard ratios with 95% confidence interval.

NAFLD cohort (n = 3613) had a median age of 43 years, 73% white, and 50% male. NAFLD group with at least one MS condition was significantly older, had higher body mass index, more likely to have insulin resistance, and heart disease compared to NAFLD group without MS. Over 19-years of follow-up, 1039 people died. Compared to NAFLD patients without MS, presence of one MS component increased the risk of mortality at 8-year (2.6% vs 4.7%) and 16-year (6% vs 11.9%) (*P* < .001). After adjusting for socio-demographic factors, NAFLD with all MS components was associated with overall, cardiac and liver-mortality. Increased number of MS components was associated with lower survival (*P* < .0001).

Patients with NAFLD and MS have higher mortality risk compared to NAFLD patients without MS. These NAFLD patients should be prioritized for the development of treatment regimens.

## 1 Introduction

Nonalcoholic fatty liver disease (NAFLD) is one of the leading causes of chronic liver disease in the world.^[1,2]^ The prevalence of NAFLD is estimated to be around 25% in the general population, and even higher in certain parts of the world and in some patient populations, such as among the obese and patients with diabetes.^[3,4]^ This high prevalence is closely associated with the rise in the rates of obesity and metabolic syndrome (MS).^[5–8]^ Components of MS include increased fasting plasma glucose or type 2 diabetes mellitus (T2DM), hypertriglyceridemia low high-density lipoprotein level, increased waist circumference, and hypertension.^[9,10,11]^. The most widely used definition of MS has been introduced by the National Cholesterol Education Program, Detection, Evaluation, and Treatment of High Blood Cholesterol in Adults, Adult Treatment Panel III (NCEP-ATP-III) guideline.^[9]^

As noted previously, there is strong evidence suggesting that components of MS are highly prevalent in patients with NAFLD. In a recent meta-analysis, the prevalence of T2DM, hypertension, and hyperlipidemia among the NAFLD cohort was 25.3%, 46.7%, and 77.1%, respectively, while 67% of subjects with NAFLD met the criteria for MS. Although the progression of NAFLD is relatively slow, given the sheer number of individuals affected by NAFLD, the clinical and economic burden of NAFLD could be enormous.^[12,13]^ In fact, currently in the United States, NAFLD is the 2nd leading indication for liver transplantation and among the top causes of hepatocellular carcinoma.^[12]^ In addition to its clinical impact, NAFLD can negatively influence economic and patient reported outcomes.^[13–15]^

Understanding which subgroups of NAFLD are especially at high risk for adverse outcomes is important. In this context, NAFLD patients with T2DM have been shown to be at greatest risk for mortality.^[16–18]^ Furthermore, a study with relatively short-term follow-up suggested that metabolically abnormal subjects with NAFLD are at higher risk of cardiac and liver mortality.^[19]^ In most studies of NAFLD, patients with NAFLD are especially at risk for cardiac mortality.^[20–22]^ In this context, it is important to determine if patients with NAFLD who are followed for a longer period of time are at risk for cardiac mortality or liver mortality, or both. Therefore, the aim of the current study is to report 19 years of follow-up for a cohort of subjects with NAFLD and assess the risk of death according to the presence of components of MS.

## 2 Methods

### 2.1 Study design and population

**Figure 1:**
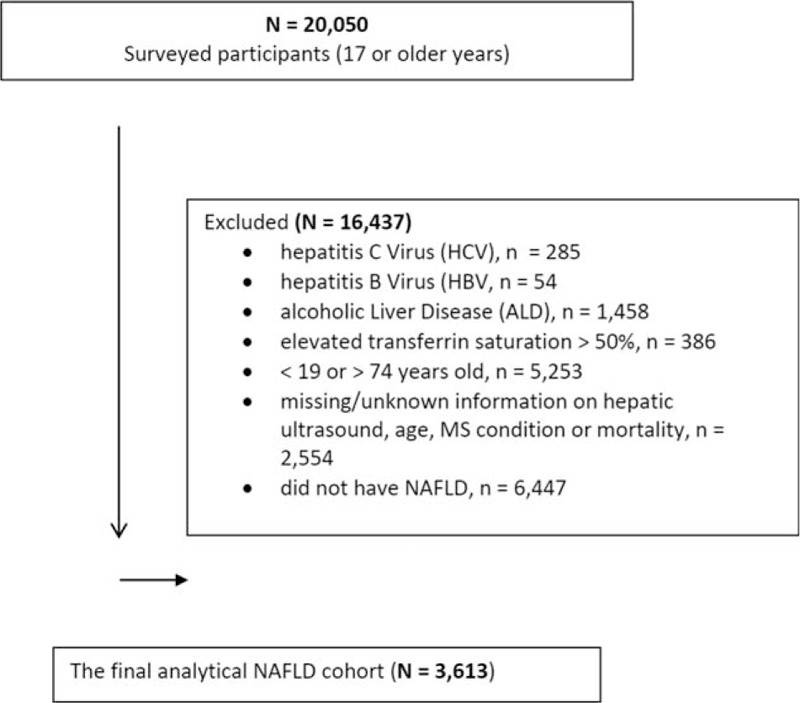
Study flow, National Health and Nutrition Examination Survey (NHANES) III.

The population of this study was obtained from the 3rd cross-sectional National Health and Nutrition Examination Survey (NHANES III), which was conducted between October 1988 and October 1994. NHANES obtained data on the health status of US civilian, noninstitutionalized population by The National Center for Health Statistics (NCHS) of the Centers for Disease Control and Prevention (CDC).^[23]^ Participants completed a self-reported demographic questionnaire (including questions about age, gender, race [White and non-White], smoking [current and past], alcohol consumption, and medical history) at home, and then underwent medical examinations performed by trained staff. Participants also provided sera (blood) samples while at the medical examination centers. Participants were included if they had a diagnosis of NAFLD as noted by hepatic ultrasound method. A Toshiba Sonolayer SSA-90A and Toshiba hepatic ultrasound video recorders were used to grade the amount of fat within the hepatic parenchyma: normal, mild, moderate, or severe steatosis. To ensure quality control, 3 ultrasound readers used standardized forms to read the ultrasounds. The readers had no access to any other participant data (see reference for more detailed information on procedure and quality assurance).^[24]^ Based on the hepatic ultrasound data, NAFLD was determined to be present if there was “mild” to “severe” hepatic steatosis and no other cause of chronic liver disease such as excessive alcohol use, elevated transferrin saturation, positive hepatitis B surface antigen (HBsAg), positive hepatitis B core antibody (anti-HBc), or positive HCV tests (anti-HCV by ELISA and HCV RNA by PCR). A grade of “normal” on the hepatic ultrasound with the absence of the above listed chronic liver disease was defined as No NAFLD, and those participants also were excluded from the final analytical cohort. Participants were also excluded if they had: hepatitis C virus (HCV) noted on their laboratory results as anti-HCV and HCV RNA confirmed as positive (more detailed information on this procedure is described elsewhere);^[25]^ hepatitis B virus (HBV) defined as being repeatedly positive for HBsAg; alcoholic liver disease (ALD) as determined if alcohol consumption was ≥20 g/day for men, ≥10 g/day for women over the past 12 months; elevated transferrin saturation >50%; were <19 or >74 years old (hepatic ultrasound imaging were performed in the age group between 20 and 74 years); missing/unknown information on hepatic ultrasound, age, MS condition, or mortality; or did not have NAFLD as a grade of “normal” finding on the hepatic ultrasound. (See cohort selection flowchart in Fig. 1. In the final analytical cohort, there were 3613 participants with NAFLD).

### 2.2 Mortality data

We obtained vital status using the Mortality Linkage File through December 31, 2011. In addition to all-cause mortality data (publically available online at https://www.cdc.gov/nchs/data-linkage/mortality-public.htm), we also used the restricted linked mortality data accessed by the Research Data Center for cause-specific mortality.^[26]^ Using the International Statistical Classification of Diseases, Injuries, and Causes of Death, 9th Revision, and the International Statistical Classification of Diseases, Injuries, and Causes of Death, 10th Revision to define the following 2 cause-specific deaths: cardiovascular-specific cause (includes major cardiovascular diseases as I00-I78); and liver-specific causes (includes viral hepatitis as B15-B19, hepatitis carcinoma as C22, and other chronic liver diseases and cirrhosis as K70; K73-K74).

### 2.3 Health conditions diagnoses and definitions

We obtained data on other medical conditions using self-reported information from the NHANES administered questionnaire questions: a history of a given health condition, use of prescription medications during a 30-day period prior to the survey, or laboratory variables. Four health conditions (diabetes, hypertension, hyperlipidemia, and central obesity) were used to identify MS as defined by NCEP-ATP-III guideline.^[9]^ Specifically, diabetes was defined as having a fasting glucose measure of ≥126 mg/dL, use of antidiabetic medication in the past month, or self-reported medical history of diabetes; hypertension was defined as having a systolic blood pressure of ≥140 mm Hg or diastolic blood pressure of ≥90 mm Hg from an average of 3 measurements, use of antihypertensive medication in the past month, or a history of high blood pressure; hyperlipidemia was defined as having high-density lipoprotein cholesterol (HDL) level of ≤40 mg/dL for men/≤50 mg/dL for women, use of antihyperlipidemia medication in the past month, or a history of hyperlipidemia; and central obesity was defined by waist circumference cut off points: for White/Black it is >102 cm for males and >88 cm for females, and for Asians it is >90 cm for males and >80 cm for females. We also identified a cardiovascular condition by the self-reported medical history of congestive heart failure, stroke, heart attack, or murmur.

### 2.4 Data analyses

All analyses were performed using SAS Version 9.3 (SAS Institute Inc.). Differences in categorical variables were evaluated by chi-square test and in mean values by *t* test according to NAFLD MS conditions. Cox proportional hazards models with linearized study design variance estimators were used to estimate hazard ratios (HRs) and their respective 95% confidence intervals (CIs) were examined the associations between MS conditions in NAFLD and all-cause and cardiovascular-specific cause outcomes. For each outcome, first we adjusted for sociodemographic (age, gender, race, smoking status, etc.) variables. Next, we adjusted for medical conditions (comorbidities, medications, and labs) using a backward model selection method to obtain the final models. Even though the number of cases for liver-related mortality was small (n = 22, 0.33%), we estimated HRs with 95% CIs (Table 4). All reported *P* values are 2-sided and defined as significant at the *P* < .05 level.

The study was approved by Inova's Institutional Review Board.

## 3 Results

Overall, the median age of the cohort was 43 years with 73% being white, and 50% being male. Table 1 shows the characteristics of the NAFLD group with at least 1 component of MS and the NAFLD group without any MS conditions. Subjects with NAFLD and at least 1 MS condition were significantly (*P* < .05) older (48 vs 35 years), had a higher body mass index (30 vs 23), were more insulin resistant (48% vs 9%), and had a higher proportion with heart disease (11% vs 1%). Approximately 11% of NAFLD with MS were taking prescribed medications for MS-related conditions of diabetes, hyperlipidemia, and hypertension. Gender and race were not associated with NAFLD in the presence of MS.

**Table 1:**
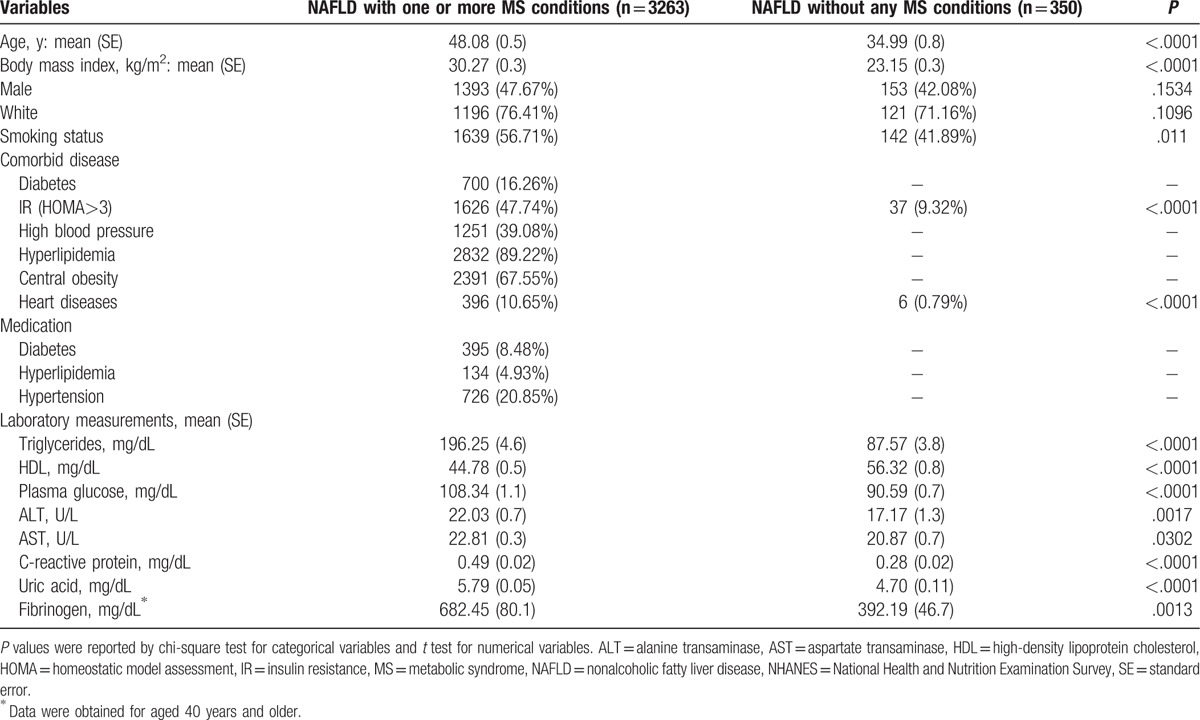
Characteristics of study by MS conditions among adult (20–74 y) participants with NAFLD, NHANES III.

### 3.1 Metabolic syndrome and overall mortality

Over an average follow-up of 19 years (IRQ = 17–21 and 71,150 person-years), there were total of 1039 deaths. The proportion of overall deaths was 34 times higher in NAFLD subjects with at least 1 component of MS than NAFLD subjects without any MS conditions (*P* < .001).

Compared to NAFLD patients without MS, presence of 1 MS component increased the risk of mortality at 8-year (2.6% vs 4.7%) and 16-year (6% vs 11.9%) (*P* < .001).

**Figure 2:**
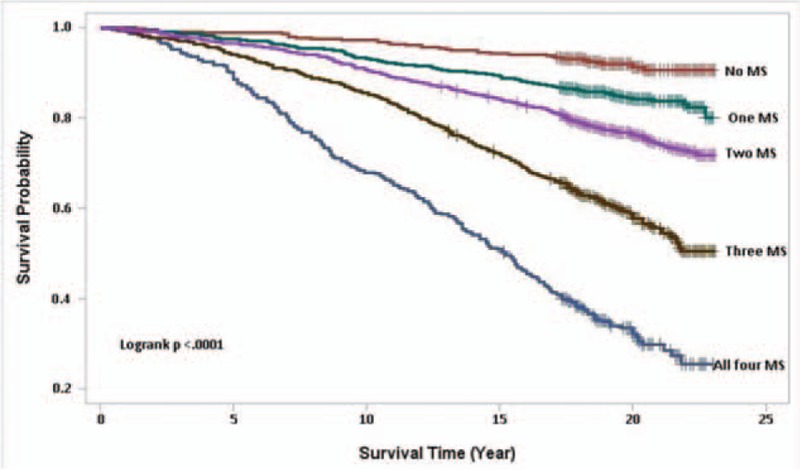
Kaplan–Meier survival curves for 23-years follow-up (average = 19 years) of 3613 NAFLD participants, by MS conditions, NHANES III, 2011 public-use linked mortality files. MS = metabolic syndrome, NAFLD = nonalcoholic fatty liver disease, NHANES = National Health and Nutrition Examination Survey.

In univariate analysis, when being compared to NAFLD subjects without any MS condition, the magnitude of the risk of death increased in NAFLD subjects with each additional MS condition (presence of 1 MS condition – HR: 1.63 [0.96–2.79]; 2 MS conditions – HR: 3.57 [2.32–5.49]; 3 MS conditions – HR: 5.87 [3.53–9.75], and all 4 MS conditions – HR: 13.09 [7.49–22.87]) (Fig. 2).

In the multivariate analysis, we examined the difference in the associations between overall mortality and each MS component after adjusting for important confounders (Table 2). Diabetes (HR: 1.78, 95% CI: 1.51–2.09) and history of heart disease (HR: 1.83, 95% CI: 1.50–2.23) were found to be the strongest independent predictors of all-cause mortality.

**Table 2:**
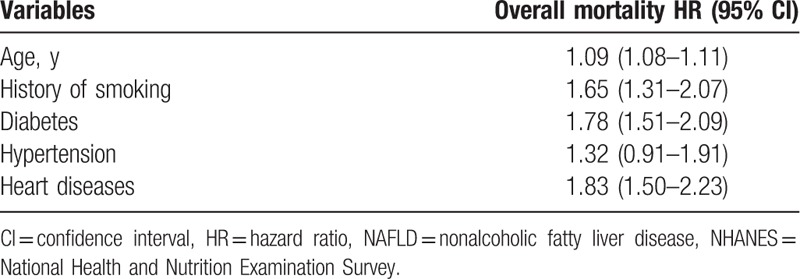
Multivariate-adjusted HRs and 95% CIs for overall mortality among adult (20–74 y) participants with NAFLD, NHANES III.

### 3.2 Metabolic syndrome and cardiac-specific cause mortality

A total of 370 cardiac-specific deaths were recorded over the 19-year follow-up period. In univariate-adjusted model, the magnitude of positive association between an increasing number of MS components and cardiovascular-specific death also increased (1 MS condition, HR: 1.02, 95% CI: 0.34–3.11; 2 MS conditions, HS: 3.32, 95% CI: 1.30–8.45; 3 MS conditions, HR: 8.03, 95% CI: 2.98–21.62; and all 4 MS conditions, HR: 16.03, 95% CI: 5.55–46.32). After adjusting for the strong confounders of cardiac death, diabetes (HR: 1.83; 95% CI: 1.35–2.47) and heart disease (HR: 2.68; 95% CI: 1.83–3.93) were the most significant predictors of cardiac-specific mortality (Table 3).

**Table 3:**
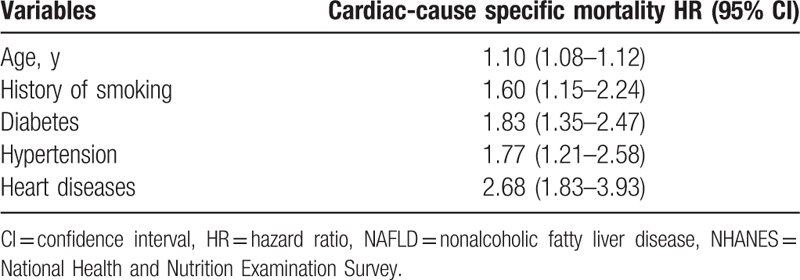
Multivariate-adjusted HRs and 95% CIs for cardiac-specific cause specific mortality among adult (20–74 y) participants with NAFLD, NHANES III.

### 3.3 Metabolic syndrome and liver-specific cause mortality

Due to a relatively small number of liver-specific mortality (n = 22), caution must be used in interpreting the results. In this context, age was the most stable predictor of the liver-specific mortality, where for each year of an increase in age, there was an 8% increase in the risk of liver-specific death (HR: 1.07; 95% CI: 1.02–1.13) (Table 4). Nevertheless, components of MS increased the risk of liver specific mortality (Table 4).

**Table 4:**
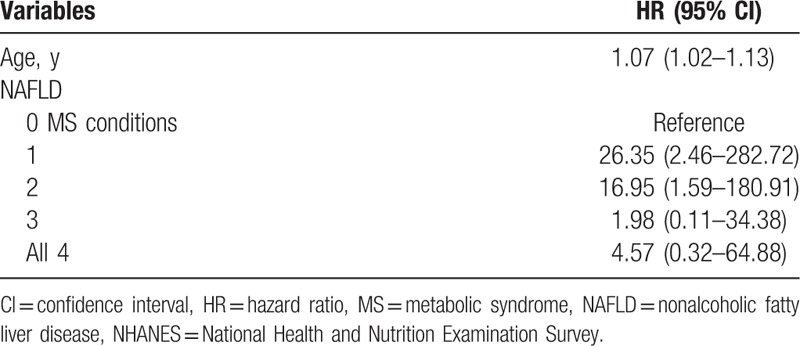
Multivariate-adjusted HRs and 95% CIs for liver-specific cause mortality among adult (20–74 y) participants with NAFLD, NHANES III.

## 4 Discussion

The association of NAFLD with MS has been widely acknowledged.^[27–29]^ In fact, many consider NAFLD as the hepatic manifestation of MS.^[30–32]^ We and others have shown that patients with NAFLD who also have DM are at increased risk for adverse outcomes.^[17,18,33,34]^ In a study with only 6 years of follow-up, we have shown that NAFLD subjects who were metabolically abnormal had higher liver and cardiac mortality.^[16,19,35–38]^ In this current NAFLD cohort with an average follow-up of 19 years, all-cause mortality was 34 times higher in NAFLD patients with components of MS than in NAFLD subjects without any MS conditions. In fact, the data suggest a graded increase in mortality with each additional component of MS. In this context, the more intense the metabolic abnormality is (as indicated by more components of MS) the higher the risk for mortality. This finding also suggests that there is an interplay between NAFLD with components of MS. In this context, presence of diabetes and hypertension were independent predictors of CV mortality and all-cause mortality.

Our findings are also in line with other reports in which cardiac disease is the most common cause of mortality in patients with NAFLD.^[39–41]^ Cardiovascular disease is frequently seen in patients with obesity.^[42–45]^ In fact, obesity is a significant risk factor for CVD a result of the interplay between hepatic insulin resistance, increased fasting glucose levels, and an atherogenic lipid profile leading to endothelial dysfunction, arterial stiffness, and myocardial dysfunction.^[42–45]^ For patients with advanced stages of NAFLD, as seen in nonalcoholic steatohepatitis, increased levels of inflammatory pro-atherogenic cytokines, hyper-coagulable factors, and adhesion molecules are also present which may lead to more severe cardiovascular disease.^[46]^ In the current study, we confirmed that with longer follow-up, cardiac mortality continued to be the dominant cause of death. Furthermore, our data indicates that components of MS, especially diabetes, is a strong predictor of cardiac mortality. As expected, presence of heart disease at the time of enrollment was the strongest predictor of cardiac mortality.

Our analysis also showed that age and having at least 1 component of MS increases the risk of liver-related mortality. In this context, diabetes and possibly dyslipidemia are known to increase the progression of liver disease.^[47,48]^

This study did have some limitations. First, since the study data are cross-sectional, no causal relationships can be established. Additionally, hepatic ultrasound for diagnosing NAFLD also has limitations. Furthermore, the reliability of self-reported alcohol consumption may be problematic. Finally, the number of subjects with liver-related mortality is relatively small (N = 22), which could have influenced the results.

In conclusion, in this study, we found that patients with NAFLD and MS had a significantly higher risk of death, especially cardiac-specific mortality compared to NAFLD patients with only 1 to 3 MS components. Given the increased risk of mortality and lower survival rates, NAFLD patients with MS components are candidates for interventional treatment. Prospective, randomized clinical studies are needed to assess the mortality benefit of the interventional therapies in these patients.

## Acknowledgments

The authors thank Brian Lam, C/PA and Beatty Liver & Obesity Research Program staff for their great support during the formation of this study.

## Author contributions

**Conceptualization:** Z.M. Younossi.

**Formal analysis:** M. Otgonsuren.

**Investigation:** M. Sayiner, N. Rafiq, P. Golabi.

**Methodology:** M. Otgonsuren, Z.M. Younossi.

**Validation:** N. Rafiq.

**Writing – original draft:** L. de Avila, M. Sayiner, P. Golabi.

**Writing – review & editing:** N. Rafiq, Z.M. Younossi.
